# OP38 Perinatal And Infant Mental Health Care: International Guideline Recommendations And Situation In Austria

**DOI:** 10.1017/S0266462324001004

**Published:** 2025-01-07

**Authors:** Ingrid Zechmeister-koss, Inanna Reinsperger, Julia Kern, Michael Edlinger, Jean Paul

## Abstract

**Introduction:**

Mental illnesses during pregnancy and after birth (perinatal period) affect one in five mothers and one in ten fathers. They can have adverse implications for child health and development and come with substantial economic costs. Many countries have developed integrated care models. We aim to summarize the international care model recommendations and contrast them with the current situation in Austria.

**Methods:**

We conducted a scoping review of guidelines and recommendations for perinatal and infant mental health care (PIMHC) models and synthesized them into a best-practice model. The model overview targets prevention, early identification, and care of perinatal mental illness (PMI), as well as professional groups involved. We identified available Austrian PIMHC services via online search and expert consultation, clustered them according to our international model structure, and visualized availability on a geographical map. Additionally, we analyzed the use of five core mental health benefits from health insurance data. We narratively contrasted international recommendations with the Austrian availability and use of services.

**Results:**

International recommendations suggest integrating primary prevention, systematic screening, and stepped care. After identifying a PMI, a care pathway needs to be defined, including diagnostic and coordinated needs-based care. For severe PMI, specialized services should be available and provided by trained psychiatrists and specialists, such as perinatal mental health midwives. In Austria, various services for different severity levels of PMI are available. However, large geographical variations and gaps exist regarding specialist services, such as mother–baby units. Specially trained health professionals are lacking. Still, almost one in five mothers claimed one of the defined mental health benefits during the perinatal period.

**Conclusions:**

PIMHC in Austria currently does not follow best-practice recommendations. The main gaps are in providing guideline-based specialized care by trained professionals. Insurance-funded mental health service use during the perinatal period corresponds with international prevalence figures, although only a part of available services is included in the analysis. To ensure women receive high-quality care, PIMHC needs to be more prioritized.

